# Fiber optic endoscopic optical coherence tomography (OCT) to assess human airways: The relationship between anatomy and physiological function during dynamic exercise

**DOI:** 10.14814/phy2.14657

**Published:** 2020-12-28

**Authors:** Carli M. Peters, Yannick Molgat‐Seon, Paolo B. Dominelli, Anthony M. D. Lee, Pierre Lane, Stephen Lam, Andrew W. Sheel

**Affiliations:** ^1^ School of Kinesiology University of British Columbia Vancouver BC Canada; ^2^ Department of Kinesiology and Applied Health University of Winnipeg Winnipeg MB Canada; ^3^ Department of Kinesiology University of Waterloo Waterloo ON Canada; ^4^ Integrative Oncology Department Imaging Unit BC Cancer Research Center Vancouver BC Canada; ^5^ School of Engineering Science Simon Fraser University Burnaby BC Canada

**Keywords:** airway size, exercise, optical coherence tomography, respiratory mechanics

## Abstract

Airway luminal area (A_i_) influences respiratory mechanics during dynamic exercise; however, previous studies have investigated the relationship between airway anatomy and physiological function in different groups of individuals. The purpose of this study was to determine the effect of A_i_ on respiratory mechanics by making *in vivo* measures of airway dimensions and work of breathing (Wb) in the same individuals. Healthy participants (3F/2M; 23–45 years) completed a cycle exercise test to exhaustion. During exercise, Wb was assessed using an esophageal balloon catheter, while simultaneously assessing minute ventilation ($\dot{V}$_E_). On a separate day, subjects underwent a bronchoscopy procedure to capture optical coherence tomography (OCT) measures of three airways in the right lung. Each participant's Wb‐$\dot{V}$_E_ data were fit to a non‐linear regression equation (Wb = *a* $\dot{V}$_E_^3^ + *b* $\dot{V}$_E_^2^) that partitions Wb into its turbulent resistive (*a*) and viscoelastic (*b*) components. Measures of A_i_ and luminal diameter were made for the 4th–6th airway generations. A composite index of airway size was calculated as the sum of the A_i_ for each generation and the total area of the 4th–6th generation was calculated based on Weibel's model. Constant *a* was significantly correlated to the Weibel model total airway area (*r* = −0.94, *p* = 0.017) and index of airway size (*r* = −0.929, *p* = 0.023), whereas constant *b* was not associated with either measure (both *p* > 0.05). We found that individuals who had the smallest A_i_ had the highest resistive Wb and our findings provide the basis for further study of the relationship between airway size and respiratory mechanics during exercise.

## Graphical abstract

We made in vivo measures of multiple airways and modeled the work of breathing (Wb)‐minute ventilation ($\dot{V}$_E_) relationship during exercise in a group of healthy young adults. We found that individuals with the highest resistive Wb had the smallest 4th–6th generation airways. These findings improve our understanding of the interrelationship between human airway dimensions and respiratory mechanics during exercise.

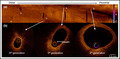

## 1 INTRODUCTION

Airway resistance is dependent on several factors including gas density and viscosity as well as airway length and radius. Of the aforementioned factors, airway radius is the most important determinant of airway resistance. During conditions where airflow is laminar, the Hagen–Poiseuille equation indicates that resistance to laminar flow is inversely proportional to the airway radius to the fourth power. When airflow becomes turbulent, airway resistance is proportional to airway diameter, as indicated by the Reynolds number equation. Based on Weibel's model, human airways branch in a dichotomous manner with each bifurcation representing one airway generation, numbered from the trachea (generation 0) to the alveoli (generation 23) (Weibel, 2001). This model assumes that each airway generation divides evenly into two approximately equal daughter airways, and although trifurcations and unevenly sized daughter airways can occur, a regular dichotomy has been demonstrated in the first six generations (Sauret et al., 2002). The progressive branching of the airways increases the total cross‐sectional area from the segmental to the terminal bronchi meaning that the larger airways (*i.e*., from the trachea to 7th generation) are the main sites of airway resistance, with airways <2 mm in diameter contributing <20% of total resistance.

Fiber optic endoscopic optical coherence tomography (OCT) is a modern imaging technique that allows three‐dimensional images of several airway generations beyond the segmental bronchi to be captured with a resolution of approximately 15 µm (Hanna et al., 2005; Huang et al., 1991). OCT‐derived measures of airway morphology may provide useful information concerning the relationship between structure and function as it relates to human airways. For example, a study investigating the relationship between structure and function in a group of current and former smokers demonstrated that OCT can detect small airway wall changes associated with a reduction in lung function in obstructive airway disease (Coxson et al., 2008).

During exercise, the mechanical work of breathing (Wb) increases exponentially as minute ventilation ($\dot{V}$_E_) rises. Utilizing esophageal pressure‐derived measures of Wb, the total Wb can be partitioned into its constituent turbulent resistive and viscoelastic components. The work done to overcome resistance to turbulent flow is reflected in the resistive component of Wb, whereas the work done to overcome the resistance of the lung tissue to deformation and of the airways to laminar flow is reflected in the viscoelastic component of Wb (Otis et al., 1950). Given the importance of airway cross‐sectional area in determining airway resistance, it can be reasoned that during exercise, when $\dot{V}$_E_ and flow increase, individuals with smaller airways would experience greater resistance to flow and therefore have a higher turbulent resistive Wb. Previously, we investigated the relationship between dysanapsis ratio, an index of airway size relative to lung size, and respiratory mechanics during exercise and demonstrated that individuals with smaller dysanapsis ratio had a higher resistive Wb (Dominelli et al., 2015). A limitation of our work was the use of an indirect measure of airway size. Determination of the effect of airway size on respiratory mechanics requires an *in vivo* evaluation of airway dimensions and quantification of Wb in the same group of subjects.

Based on the above summary, the purpose of this study was to determine the relationship between measures of airway area and the resistive Wb in healthy individuals under conditions of dynamic exercise where $\dot{V}$_E_ is high. To do so, we applied a novel imaging modality to obtain an *in vivo* evaluation of multiple airway generations coupled with a detailed assessment of pulmonary mechanics in healthy humans. We hypothesized that subjects with the smallest airway area would have the highest resistive Wb for a given $\dot{V}$_E_.

## 2 METHODS

### 2.1 Ethical approval

This study was approved by the Clinical Research Ethics Board at the University of British Columbia (approval number: H14‐00724) and conformed to the standards set by the *Declaration of Helsinki*, except for registration in a database. All participants provided written informed consent.

### 2.2 Participants

Healthy females (*n* = 3) and males (*n* = 2) between the ages of 23–45 years with normal pulmonary function based on predicted values (Quanjer et al., 2012; Stocks & Quanjer, 1995) were included.

### 2.3 Experimental protocol

The study took place over 2 days. Day 1 consisted of pulmonary function testing followed by a cycle exercise test to exhaustion, while instrumented with an esophageal balloon catheter to determine the components of Wb. On day 2, subjects underwent a bronchoscopy procedure to capture OCT images of multiple airways. Measures of airway luminal area (A_i_) and mean luminal diameter were made for the 4th–6th airway generations (Kirby et al., 2015).

### 2.4 General procedures

Spirometry and plethysmography were performed using a commercially available system (Vmax Encore 229, V62J Autobox; CareFusion) according to standard recommendations. The incremental cycle test to exhaustion was performed on an electromagnetically braked ergometer. Participants began at a work rate of 80 W (women) or 120 W (men) and work increased every 2 min by 20 W for both sexes until volitional exhaustion. Flows, volume, and esophageal pressures (balloon‐tipped catheter) were obtained using previously described methods (Dominelli et al., 2015). Following exercise, subjects completed forced vital capacity (FVC) maneuvers to ensure forced expired volume in 1 s (FEV_1_) was not reduced during exercise. All raw data collected on the first day of testing were recorded at 200 Hz continuously using a 16‐channel analog‐to‐digital data acquisition system (PowerLab/16SP model ML 795, ADIinstrument, Colorado Springs, CO) and stored on a personal computer for analysis.

### 2.5 Work of breathing

Wb was determined as previously described (Dominelli & Sheel, 2012), whereby the area of esophageal pressure–volume curves generated by composite averaging pressure and volume data over 30 s of rest and each 2 min exercise stage was integrated. The Wb–$\dot{V}$_E_ relationship across a range of ventilations was then modeled by fitting each participants’ data to the following equation (Otis et al., 1950):

(1) $$\text{Wb}=a{\dot{V}}_{E}^{3}+b{\dot{V}}_{E}^{2}$$

Where *a* $\dot{V}$_E_^3^ represents turbulent resistive Wb and *b* $\dot{V}$_E_^2^ represents viscoelastic Wb.

### 2.6 OCT imaging and measurements

**FIGURE 1:**
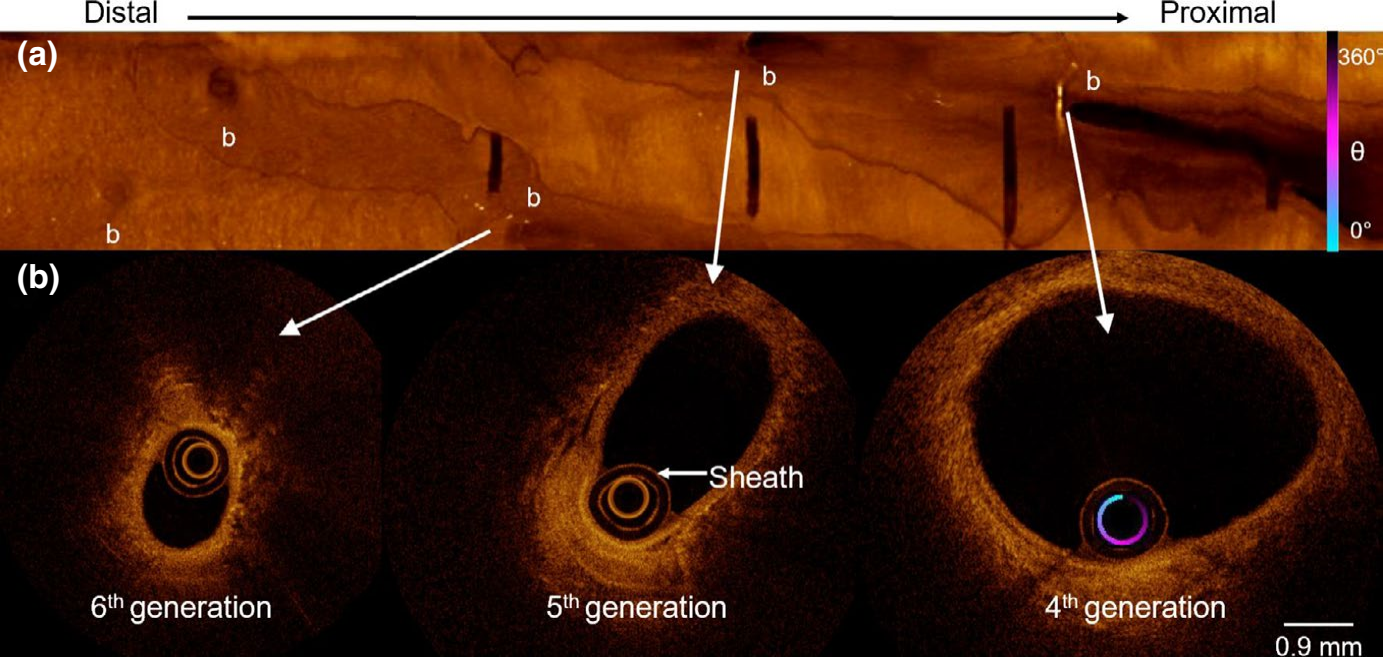
Panel (a) Mean intensity en face projection optical coherence tomography image of RB9 from Female 1. From left to right, the pullback is shown from distal to proximal. Branch points are marked with a b. (b) From left to right, cross‐sectional images of the sixth, fifth, and fourth‐generation bronchi

Bronchoscopy procedures were performed at the British Columbia Cancer Agency under local anesthesia and conscious sedation. An in‐house built 0.9 mm diameter OCT rotary‐pullback catheter was inserted through the biopsy channel of the bronchoscope into segmental airways in the upper and lower lobes of the right lung. The catheter was advanced until the internal diameter of the airway was equal to the outer diameter of the catheter. A 7 cm pullback distal to the segmental bronchi was imaged in the upper lobe (RB2 or RB3) and two 7 cm pullbacks were imaged in the lower lobe (RB8 and RB9). OCT images were acquired using an OCT system driven by a 50 kHz swept‐source laser (SSOCT‐1310, Axsun Technologies Inc., Billerica, Massachusetts). The pullback speed was set to 5 mm s^−1^ and the probe rotated at 49 Hz. The generation of bronchi was determined by counting airway branches from the subsegmental entrance in movie exports of the airway pullbacks (Video S1). Branches are indicated in the mean intensity en face projection shown in Figure 1. Airway measures included the average lumen diameter and A_i_ from the 4th–6th generation of bronchi (Kirby et al., 2015). Airway measures were made using customized software. Measures of A_i_ of the 4th–6th generation from the three pullbacks were averaged. An index of airway size was calculated as the sum of the 4th–6th generation A_i_ for each participant. A total cross‐sectional area for the 4th–6th generations was calculated based on the branching pattern modeled by Weibel (2001). The number of airways in each generation was approximated by the number 2 raised to the power of generation number such that there are 16, 32, and 64 airways in the fourth, fifth, and sixth generations, respectively.

### 2.7 Statistics

The Wb–$\dot{V}$_E_ relationship for each participant was fit to Equation 1. Pearson product‐moment correlations were used to determine linear relationships between selected dependent variables. Statistical analysis was performed using SigmaPlot software. The level of statistical significance was set at *p* < 0.05. All data are presented as means ± standard deviation.

## 3 RESULTS

### 3.1 Participants

Participant characteristics, pulmonary function, and maximal exercise data are presented in Table 1.

**TABLE 1:** Participant characteristics, pulmonary function, and maximal exercise data

	Subject 1	Subject 2	Subject 3	Subject 4	Subject 5
Subject characteristics					
Sex	Female	Female	Female	Male	Male
Age, y	24	25	23	27	45
Height, cm	171	170	163	172	175
Mass, kg	58.8	67.5	57.6	73.0	71.8
Resting pulmonary function					
FVC, l (%predicted)	4.62 (104%)	4.41 (101%)	4.03 (100%)	6.33 (123%)	5.11 (104%)
FEV_1_, l (%predicted)	3.49 (94%)	3.71 (101%)	3.26 (94%)	4.6 (107%)	3.63 (90%)
TLC, l (%predicted)	6.28 (105%)	5.70 (96%)	5.03 (92%)	7.97 (116%)	7.74 (115%)
VC, l (%predicted)	4.84 (109%)	4.41 (101%)	4.21 (104%)	6.48 (122%)	5.34 (108%)
PEF, l·s^−1^ (%predicted)	7.96 (107%)	6.67 (90%)	7.54 (109%)	11.04 (113%)	10.38 (116%)
FEF_25−75_, l·s^−1^	2.81 (74%)	4.11 (109%)	3.02 (82%)	3.52 (81%)	2.45 (88%)
FEF_25_, l·s^−1^	7.13 (115%)	6.61 (108%)	6.84 (116%)	7.49 (95%)	7.58 (92%)
FEF_50_, l·s^−1^	3.64 (79%)	4.92 (107%)	3.64 (78%)	4.23 (75%)	3.51 (72%)
FEF_75_, l·s^−1^	1.32 (65%)	2.21 (109%)	1.56 (74%)	1.79 (77%)	0.88 (45%)
Maximal exercise data					
V_T_, l	1.8	2.2	1.4	3.2	2.4
F_b,_ b·min^−1^	55	48	70	44	52
$\dot{V}$_E_, l·min^−1^	102	106	100	143	126
$\dot{V}$ O_2_, l·min^−1^	3.1	2.9	2.5	3.6	4.2
Wb, J·min^−1^	283	425	343	511	419
Workload, W	240	200	220	260	340

Abbreviations: $\dot{V}$_E_, minute ventilation; Fb, breathing frequency; FEF_25_, forced expired flow after 25% volume expired; FEF_50_, forced expired flow after 50% volume expired; FEF_75_, forced expired flow after 75% volume expired; FEV_1_, forced expired volume in 1 s; FVC, forced vital capacity; $\dot{V}$O_2_, oxygen consumption; PEF, peak expiratory flow; TLC, total lung capacity; VC, vital capacity; V_T_, tidal volume; Wb, work of breathing.

### 3.2 OCT airway measures

**FIGURE 2:**
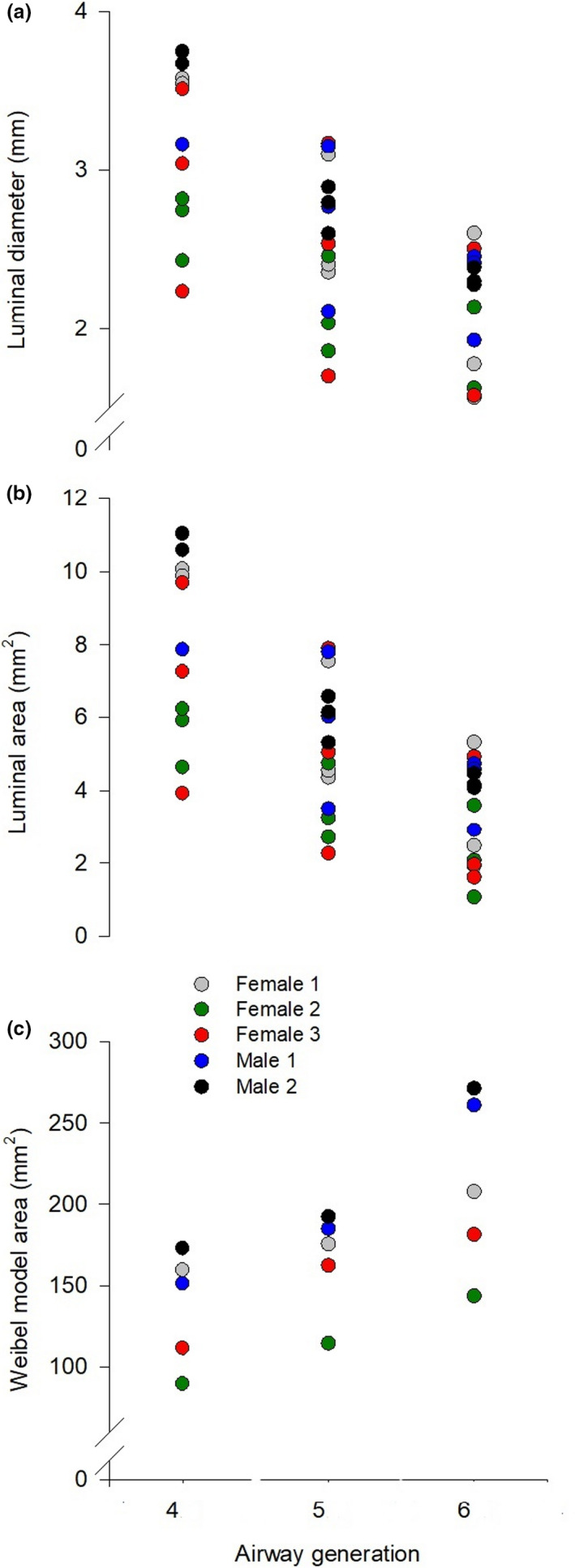
Luminal diameter (Panel a) and luminal area (Panel b) of the fourth, fifth, and sixth generation bronchi for each subject for all three airways imaged and the Weibel model area (Panel c) of the fourth, fifth, and sixth generation

Three measures of 4th–6th generation airways were made in subjects 2 and 3. In subjects 1, 4, and 5, the fourth‐generation airway was not visible in one of the three pullbacks. As such, two measures of the fourth‐generation airway and three measures of the fifth and sixth generation airways were made in these subjects. Individual luminal diameter, A_i_, and Weibel model area measures for each subject are shown in Figure 2. The average luminal diameter of the fourth, fifth, and sixth‐generation airways was 3.3 ± 0.4 mm, 2.6 ± 0.3 mm, and 2.0 ± 0.3 mm, respectively. The average A_i_ of the 4th–6th generation airways was 8.6 ± 2.2 mm^2^, 5.2 ± 1.0 mm^2^, and 3.3 ± 0.8 mm^2^, respectively. Airway area calculated based on the Weibel's model increased from the fourth through sixth generation (137 ± 35 mm^2^ to 213 ± 54 mm^2^).

### 3.3 Relationship between physiological variables and airway measures

**FIGURE 3:**
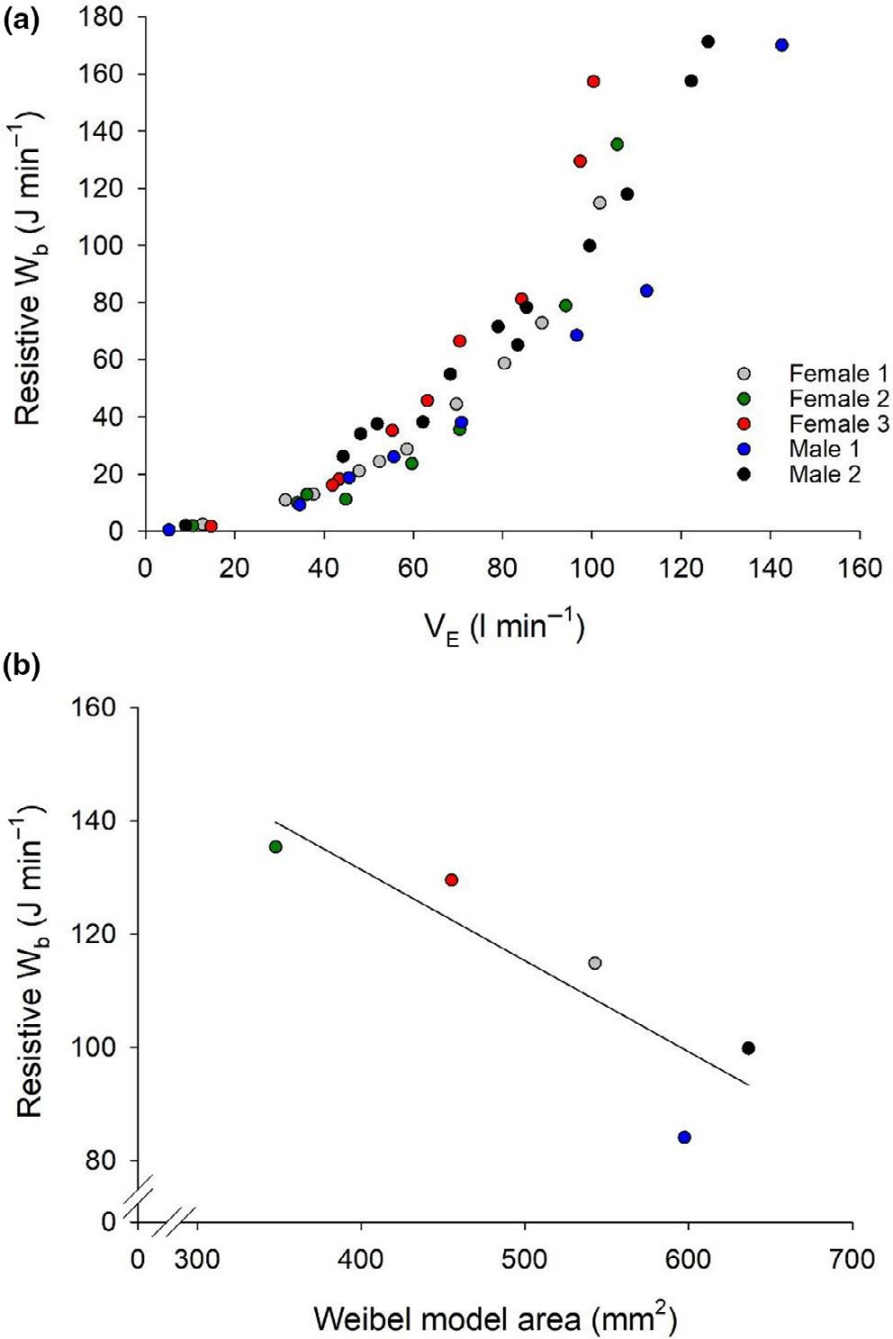
(a) The relationship between resistive work of breathing (Wb) and minute ventilation ($\dot{V}$_E_) during exercise. Individual data for each stage of incremental exercise is presented for all subjects. (b) Shows the relationship between the Weibel model area for each individual and their resistive Wb at the highest equivalent $\dot{V}$_E_ (100 ± 4 L·min^−1^, *r* = −0.897, *p* = 0.039)

**FIGURE 4:**
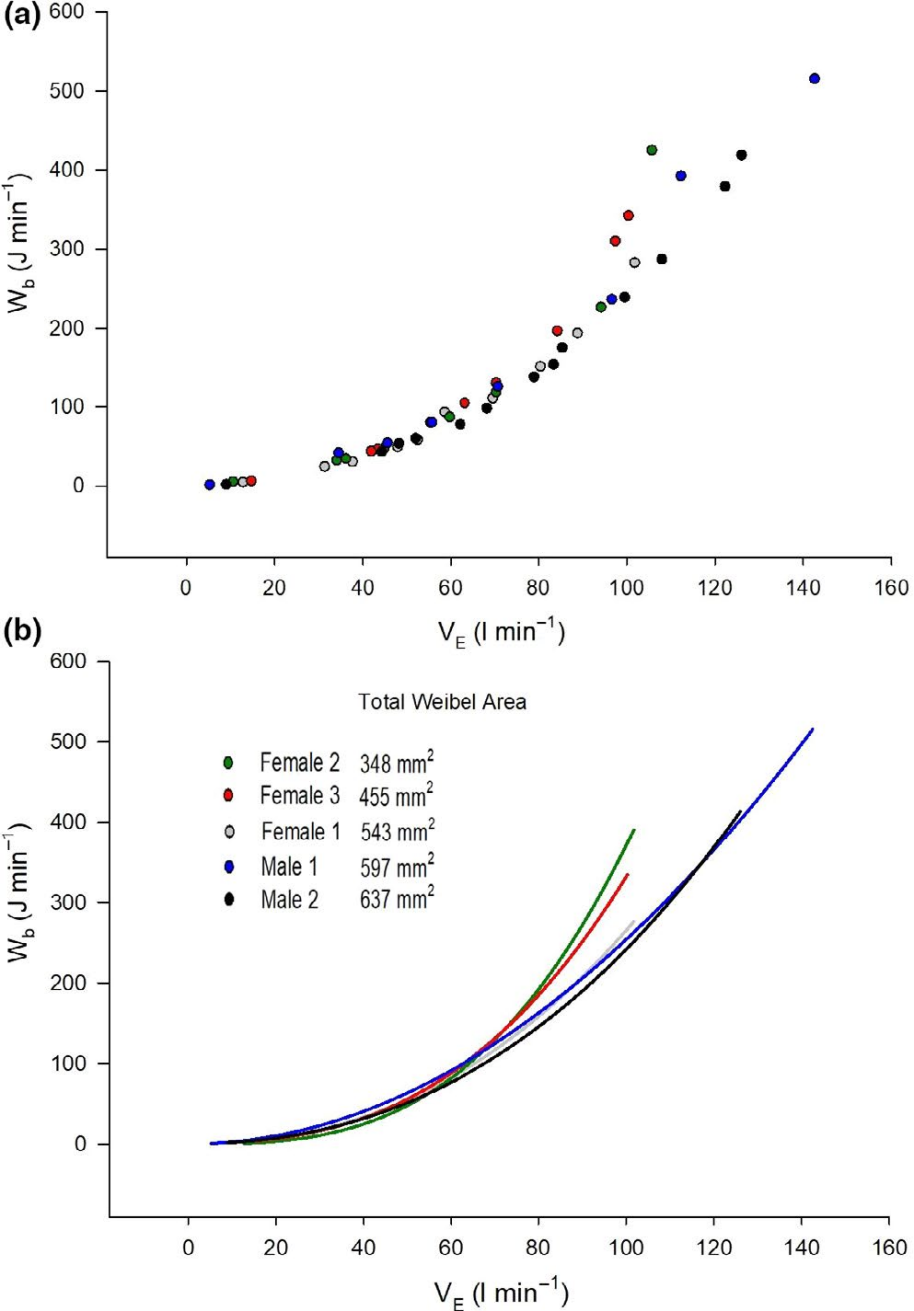
The relationship between total work of breathing (Wb) and minute ventilation ($\dot{V}$_E_) during exercise. Panel (a) shows individual data for each stage of incremental exercise. Panel (b) shows individual curves based on values of constants *a* and *b* from Equation 1. Note that subjects have been listed in order of increasing the Weibel model area and that Wb curves closely follow this order with the female with the smallest Weibel area having the highest Wb for a given $\dot{V}$_E_

At a moderate $\dot{V}$_E_ (70 ± 1 L min^−1^) there was no relationship between the resistive Wb and the Weibel model total airway area (*r* = 0.151, *p* = 0.81), whereas at a high $\dot{V}$_E_ (100 ± 4 L min^−1^) there was a significant relationship (*r* = −0.883, *p* = 0.047) (Figure 3). Each participant's Wb–$\dot{V}$_E_ curve was fit to Equation 1 and the mean *r^2^* was 0.99 ± 0.01 (Figure 4). Mean values for constants *a* and *b* were 1.33 ± 1.05.10^–4^ and 1.51.10^–2^ ± 6.99.10^–3^, respectively. Constant *a* was significantly correlated to the Weibel model total airway area (*r* = −0.94, *p* = 0.017), index of airway size (*r* = −0.929, *p* = 0.023), and PEF (*r* = −0.897, *p* = 0.039). Constant *b* was not significantly associated with any variables in this study.

## 4 DISCUSSION

### 4.1 Major findings

The major finding of this study is that OCT measured airway size across multiple generations is significantly associated with Wb during exercise in healthy individuals. Our results, which are not confounded by smoking history or indirect measures of airway anatomy, demonstrate that individuals with the smallest 4th–6th generation airways have the highest Wb and that the increased Wb can be attributed to a higher turbulent resistive component. Our findings extend our understanding of how airway size is an important determinant of respiratory mechanics during exercise.

### 4.2 Relationship between airway size and Wb

Fiber optic OCT is a contemporary imaging modality used in pulmonary medicine. Previous work suggests a significant correlation between OCT measures of airway morphology compared with computed tomography (CT) (Coxson et al., 2008) and histopathological assessments (Lee et al., 2014). What makes OCT particularly appealing for studying healthy individuals is the lack of ionizing radiation exposure compared to CT (Brenner & Hall, 2007) and the ability not to alter or remove tissue compared to biopsy studies. Recently, OCT measures of human airways were validated by comparison with chest CT and pathology up to the 9th generation bronchi (Chen et al., 2015). Promisingly, the luminal diameter of the 4th–6th generation airways in our study is very similar to a recently published study investigating normal airway morphology with OCT in a large cohort (Su et al., 2019).

As airway size is related to lung size and height, taller individuals are likely to have larger airways; however, there is considerable variability between individuals (Dominelli et al., 2018). Variability in airway size and its impact on pulmonary mechanics has important physiological implications. From rest to maximal exercise, the mechanical Wb increases exponentially as a function of $\dot{V}$_E_ (Otis et al., 1950). Our findings suggest that smaller 4th–6th generation airways contribute to a disproportionate rise in the resistive Wb when $\dot{V}$_E_ is high (Figure 3). We are confident that the increased resistive Wb in subjects with smaller airways is not due to exercise‐induced bronchoconstriction as none of the subjects had been previously diagnosed with asthma and FEV_1_ measured immediately after exercise did not differ from baseline values. An increased Wb for a given $\dot{V}$_E_ may predispose individuals with smaller airways to pulmonary system limitations to exercise, such as expiratory flow limitation and respiratory and locomotor muscle fatigue (Dempsey et al., 2008).

The purpose of this study was not to investigate sex‐based differences in airway size on Wb, but our observations in male and female participants merit brief discussion. We have previously shown that women have a higher resistive Wb during exercise compared to men and have suggested that smaller airways are a causative factor (Dominelli et al., 2015; Molgat‐Seon et al., 2019). Females 2 and 3 had smaller airways than both male subjects; however, female 1’s airways were similarly sized to both males (Figure 2). Females 1 and 2 are of similar age (24 vs. 25 years old) and height (171 vs. 170 cm), yet female 1 had a much larger calculated total airway area (543 vs. 348 mm^2^). Variability within and between sexes with respect to airway size is consistent with previously published CT airway measures in healthy subjects and smokers (Dominelli et al., 2018; Sheel et al., 2009). Future studies with a larger sample size of men and women are required to further our understanding of how innate sex‐differences in anatomy may affect respiratory mechanics during exercise.

### 4.3 Limitations

Several limitations associated with our study should be acknowledged. First, we recognize that the small sample size limits the generalizability of our findings. We have been careful not to overstate our findings and we acknowledge the limitations of our sample size and the limits of correlative evidence. However, the novelty of our findings—application of highly invasive procedures to ascertain airway dimensions—sets the stage for future investigations aimed at understanding the relationships between human airway anatomy and pulmonary mechanics. Second, because of the imaging system used, we were unable to measure the larger conducting airways that also contribute to differences in resistance. However, the 4th–6th generation airway size differences are likely representative of individual size differences in the larger conducting airways. Third, the airway luminal area can vary depending on lung volume owing to radial traction on the airways. In our study, subjects were asked to hold their breath at the end of a full inspiration (*i.e*., total lung capacity) during the pullback OCT procedure, which lasted for approximately 14 s. In some instances, participants had a difficult time holding their breath at TLC with conscious sedation and began tidally breathing prior to the pullback being complete. This may have influenced our findings; however, we have no data which speak directly to this possibility nor the magnitude of any potential effect.

## 5 CONCLUSIONS

Utilizing a contemporary imaging method, OCT, we have provided new insight into our understanding of pulmonary mechanics in exercising humans. We found that individuals with the smallest airways had the highest Wb at levels of high $\dot{V}$_E_ and that the increased Wb could be explained by a larger resistive Wb. Our findings provide the basis for further study of the interrelationship between healthy human airway dimensions and respiratory mechanics during high‐intensity exercise.

## CONFLICT OF INTEREST

The authors have no conflicts of interest to disclose.

## AUTHOR CONTRIBUTIONS

CMP, YMS, PBD, AMDL, PL, SL, and AWS designed the study. CMP, YMS, PBD, and AMDL enrolled participants, conducted data collection, and analyzed the data. All authors had complete access to all the study data, contributed to drafting and critically revising the manuscript. All authors approved the final version of the manuscript and take responsibility for the integrity of the data and the accuracy of the data analysis.

## Supporting information



Video S1

## References

[phy214657-bib-0001] Brenner, D. J. , & Hall, E. J. (2007). Computed tomography–an increasing source of radiation exposure. The New England Journal of Medicine, 357, 2277–2284. 10.1056/NEJMra072149 18046031

[phy214657-bib-0002] Chen, Y. U. , Ding, M. , Guan, W.‐J. , Wang, W. , Luo, W.‐Z. , Zhong, C.‐H. , Jiang, M. , Jiang, J.‐H. , Gu, Y.‐Y. , Li, S.‐Y. , & Zhong, N.‐S. (2015). Validation of human small airway measurements using endobronchial optical coherence tomography. Respiratory Medicine, 109, 1446–1453. 10.1016/j.rmed.2015.09.006 26427628

[phy214657-bib-0003] Coxson, H. O. , Quiney, B. , Sin, D. D. , Xing, L. , McWilliams, A. M. , Mayo, J. R. , & Lam, S. (2008). Airway wall thickness assessed using computed tomography and optical coherence tomography. American Journal of Respiratory and Critical Care Medicine, 177(11), 1201–1206. 10.1164/rccm.200712-1776OC 18310475 PMC2408438

[phy214657-bib-0004] Dempsey, J. A. , Miller, J. D. , Romer, L. , Amann, M. , & Smith, C. A. (2008). Exercise‐induced respiratory muscle work: Effects on blood flow, fatigue and performance. Advances in Experimental Medicine and Biology, 605, 209–212. 18085273 10.1007/978-0-387-73693-8_36 PMC12869849

[phy214657-bib-0005] Dominelli, P. B. , Molgat‐Seon, Y. , Bingham, D. , Swartz, P. M. , Road, J. D. , Foster, G. E. , & Sheel, A. W. (2015). Dysanapsis and the resistive work of breathing during exercise in healthy men and women. Journal of Applied Physiology, 119, 1105–1113. 10.1152/japplphysiol.00409.2015 26359483 PMC4816413

[phy214657-bib-0006] Dominelli, P. B. , Ripoll, J. G. , Cross, T. J. , Baker, S. E. , Wiggins, C. C. , Welch, B. T. , & Joyner, M. J. (2018). Sex differences in large conducting airway anatomy. Journal of Applied Physiology, 125, 960–965. 10.1152/japplphysiol.00440.2018 30024341 PMC6335094

[phy214657-bib-0007] Dominelli, P. B. , & Sheel, A. W. (2012). Experimental approaches to the study of the mechanics of breathing during exercise. Respiratory Physiology & Neurobiology, 180, 147–161. 10.1016/j.resp.2011.10.005 22019486

[phy214657-bib-0008] Hanna, N. , Saltzman, D. , Mukai, D. , Chen, Z. , Sasse, S. , Milliken, J. , Guo, S. , Jung, W. , Colt, H. , & Brenner, M. (2005). Two‐dimensional and 3‐dimensional optical coherence tomographic imaging of the airway, lung, and pleura. The Journal of Thoracic and Cardiovascular Surgery, 129, 615–622. 10.1016/j.jtcvs.2004.10.022 15746746

[phy214657-bib-0009] Huang, D. , Swanson, E. , Lin, C. , Schuman, J. , Stinson, W. , Chang, W. , Hee, M. , Flotte, T. , Gregory, K. , Puliafito, C. , & et, A. L. (1991). Optical coherence tomography. Science, 254, 1178–1181. 10.1126/science.1957169 1957169 PMC4638169

[phy214657-bib-0010] Kirby, M. , Ohtani, K. , Nickens, T. , Lisbona, R. M. L. , Lee, A. M. D. , Shaipanich, T. , Lane, P. , MacAulay, C. , Lam, S. , & Coxson, H. O. (2015). Reproducibility of optical coherence tomography airway imaging. Biomedical Optics Express, 6, 4365–4377. 10.1364/BOE.6.004365 26601002 PMC4646546

[phy214657-bib-0011] Lee, A. M. D. , Kirby, M. , Ohtani, K. , Candido, T. , Shalansky, R. , MacAulay, C. , English, J. , Finley, R. , Lam, S. , Coxson, H. O. , & Lane, P. (2014). Validation of airway wall measurements by optical coherence tomography in porcine airways. PLoS One, 9, e100145 10.1371/journal.pone.0100145 24949633 PMC4064993

[phy214657-bib-0012] Molgat‐Seon, Y. , Dominelli, P. B. , Guenette, J. A. , & Sheel, A. W. (2019). Modelling the effects of age and sex on the resistive and viscoelastic components of the work of breathing during exercise. Experimental Physiology, 104, 1737–1745. 10.1113/EP087956 31408911

[phy214657-bib-0013] Otis, A. B. , Fenn, W. O. , & Rahn, H. (1950). Mechanics of breathing in man. Journal of Applied Physiology, 2, 592–607. 15436363 10.1152/jappl.1950.2.11.592

[phy214657-bib-0014] Quanjer, P. H. , Stanojevic, S. , Cole, T. J. , Baur, X. , Hall, G. L. , Culver, B. H. , Enright, P. L. , Hankinson, J. L. , Ip, M. S. M. , Zheng, J. , & Stocks, J. (2012). Multi‐ethnic reference values for spirometry for the 3–95‐yr age range: The global lung function 2012 equations. The European Respiratory Journal, 40, 1324–1343. 10.1183/09031936.00080312 22743675 PMC3786581

[phy214657-bib-0015] Sauret, V. , Halson, P. M. , Brown, I. W. , Fleming, J. S. , & Bailey, A. G. (2002). Study of the three‐dimensional geometry of the central conducting airways in man using computed tomographic (CT) images. Journal of Anatomy, 200, 123–134. 10.1046/j.0021-8782.2001.00018.x 11895110 PMC1570625

[phy214657-bib-0016] Sheel, A. W. , Guenette, J. A. , Yuan, R. , Holy, L. , Mayo, J. R. , McWilliams, A. M. , Lam, S. , & Coxson, H. O. (2009). Evidence for dysanapsis using computed tomographic imaging of the airways in older ex‐smokers. Journal of Applied Physiology, 107, 1622–1628. 10.1152/japplphysiol.00562.2009 19762522 PMC2777797

[phy214657-bib-0017] Stocks, J. , & Quanjer, P. H. (1995). Reference values for residual volume, functional residual capacity and total lung capacity. ATS Workshop on Lung Volume Measurements. Official Statement of The European Respiratory Society. The European Respiratory Journal, 8, 492–506. 10.1183/09031936.95.08030492 7789503

[phy214657-bib-0018] Su, Z.‐Q. , Guan, W.‐J. , Li, S.‐Y. , Feng, J.‐X. , Zhou, Z.‐Q. , Chen, Y. U. , Zhong, M.‐L. , & Zhong, N.‐S. (2019). Evaluation of the normal airway morphology using optical coherence tomography. Chest, 156, 915–925. 10.1016/j.chest.2019.06.009 31265836

[phy214657-bib-0019] Weibel, E. R. (2001). Why measure lung structure? American Journal of Respiratory and Critical Care Medicine, 163, 314–315. 10.1164/ajrccm.163.2.hh12-00 11179098

